# Responding to Climate and Environmental Change Impacts on Human Health via Integrated Surveillance in the Circumpolar North: A Systematic Realist Review

**DOI:** 10.3390/ijerph15122706

**Published:** 2018-11-30

**Authors:** Alexandra Sawatzky, Ashlee Cunsolo, Andria Jones-Bitton, Jacqueline Middleton, Sherilee L. Harper

**Affiliations:** 1 Department of Population Medicine, University of Guelph, 50 Stone Road E, Guelph, ON N1G 2W1, Canada; aqjones@uoguelph.ca (A.J.-B.); jmiddl03@uoguelph.ca (J.M.); 2 Labrador Institute of Memorial University, 219 Hamilton River Road, P.O. Box 490, Stn. B, Happy Valley-Goose Bay, NL A0P 1E0, Canada; 3 School of Public Health, University of Alberta, 116 St. and 85 Ave., Edmonton, AB T6G 2R3, Canada

**Keywords:** circumpolar North, climate change, adaptation, environmental health, public health, surveillance

## Abstract

Environments are shifting rapidly in the Circumpolar Arctic and Subarctic regions as a result of climate change and other external stressors, and this has a substantial impact on the health of northern populations. Thus, there is a need for integrated surveillance systems designed to monitor the impacts of climate change on human health outcomes as part of broader adaptation strategies in these regions. This review aimed to identify, describe, and synthesize literature on integrated surveillance systems in Circumpolar Arctic and Subarctic regions, that are used for research or practice. Following a systematic realist review approach, relevant articles were identified using search strings developed for MEDLINE^®^ and Web of Science™ databases, and screened by two independent reviewers. Articles that met the inclusion criteria were retained for descriptive quantitative analysis, as well as thematic qualitative analysis, using a realist lens. Of the 3431 articles retrieved in the database searches, 85 met the inclusion criteria and were analyzed. Thematic analysis identified components of integrated surveillance systems that were categorized into three main groups: structural, processual, and relational components. These components were linked to surveillance attributes and activities that supported the operations and management of integrated surveillance. This review advances understandings of the distinct contributions of integrated surveillance systems and data to discerning the nature of changes in climate and environmental conditions that affect population health outcomes and determinants in the Circumpolar North. Findings from this review can be used to inform the planning, design, and evaluation of integrated surveillance systems that support evidence-based public health research and practice in the context of increasing climate change and the need for adaptation.

## 1. Introduction

Arctic and Subarctic regions across the Circumpolar North are experiencing some of the most dramatic and rapid environmental changes in the world, largely due to unprecedented climate change and variation. Climate change is associated with rising atmospheric temperatures, increases in extreme weather events and storms, and warming permafrost [1,2,3,4,5,6], and is compounded by other large-scale drivers of socio-ecological change, such as natural resource development [7,8]. Furthermore, the impacts of climate change in the Circumpolar North are highly complex, varying in rate and magnitude by region and by season [3,9].

Increasing climate change and variation is also creating new challenges for the health of northern populations [6,9,10,11,12]. For example, rising temperatures have disrupted ice formation and breakup patterns, leading to unsafe and unpredictable travel conditions and, in turn, have increased rates of injuries and death [13,14,15,16,17]. Fluctuations in ice safety and weather patterns can disrupt the ability to hunt, leading to decreased food security and nutritional deficits [18,19,20,21,22]. Extreme weather events and changes in precipitation patterns can increase run-off and contaminate local water supplies, increasing the risk of waterborne disease and acute gastrointestinal illness [19,23]. Also, the ongoing stress and uncertainty related to changing climatic and environmental conditions can lead to negative impacts on mental wellness [24,25,26].

In recent years, public health surveillance systems have been identified as important tools for characterizing the burden and distribution of human health outcomes associated with climate change and related environmental shifts [7,27,28,29,30]. Some existing public health surveillance systems in the Circumpolar North have documented associations between various environmental hazards and human health outcomes [27,31,32]. These systems, however, are often not designed for, nor are adequately equipped to detect and respond to multiple sources of variability and change in the environment, and nor are they structured to understand the cumulative nature of climate-sensitive health outcomes [33]. Consequently, there is a lack of appropriate methods for collecting, analyzing, and interpreting surveillance data for public health decision-making and responses to health issues that are of particular concern in the Arctic and Subarctic regions [12,19,34,35]. In effect, this leads to gaps in understandings of how increasing climate change might affect population health in the North in the future [12,35]. Addressing these gaps will require improved coordination over the development and implementation of surveillance systems in northern regions, and globally, that integrate considerations for human health in the context of rapid climate change [8,32].

Integrated surveillance systems are designed to monitor and enable responses to one or more aspect(s) of the natural environment, and associated impacts on one or more human health outcome(s) to monitor trends and identify opportunities for public health responses [36,37,38]. Such systems can serve as important tools for integrating different types and sources of data that help further explain and contextualize the range, nature, and extent of potential interactions between environmental hazards, human exposure, and health outcomes [39,40]. For populations in the Arctic and Subarctic regions, interactions between climate change and other environmental, cultural, social, economic, and political factors can lead to public health challenges that differ substantially from those of their southern counterparts [27]. In these northern regions, integrated surveillance systems can generate data to inform more comprehensive, targeted responses to particular public health challenges in the context of climate change that may be outside the scope or capability of other types of surveillance systems [8].

The current status of environment or health surveillance in the North has been explored in several national and international reports [8,27,34,41], and some studies have reviewed the contributions of other forms of surveillance systems to addressing environmental or public health issues in the North [42,43]. However, few studies have systematically reviewed and synthesized information from the academic literature about existing *integrated* environment *and* health surveillance systems designed for Arctic and Subarctic regions. In addition, although the attributes and components of many types of public health surveillance systems have been characterized and extensively reviewed elsewhere [44,45,46], limited research has examined the attributes and components of integrated surveillance systems designed specifically for the dynamic, interconnected environmental and human health issues in Northern regions, and none in the context of climate change. This presents an opportunity to further understandings on how components that comprise a surveillance system can enable and enhance certain surveillance attributes and activities (i.e., the collection, analysis, interpretation, and communication of environment and health data), to direct and target appropriate public health action and responses to climate change in the North. A synthesis and critical analysis of this literature can help guide the identification of opportunities for designing, implementing, and evaluating integrated surveillance systems. Taking advantage of these opportunities will be important for addressing distinct local, regional, national, and international public health priorities at the intersection of environmental and human health in the North, and in the context of climate change and other large-scale socio-ecological drivers of change. Therefore, this research aimed to identify, describe, and synthesize academic literature on integrated environment and health surveillance systems in Circumpolar Arctic and Subarctic regions, that are used for research or practice. Within this goal were two main objectives: (i) to provide an overview of the range, distribution, and attributes of integrated environment and health surveillance systems used for research or practice in the Arctic and Subarctic that are discussed in the literature; (ii) to compare, contrast, and characterize the enabling components for integrated system surveillance development, implementation, and use in these regions. 

## 2. Materials and Methods

This research followed a systematic realist review approach, applying a systematic process for searching and identifying relevant literature by using transparent, replicable methods [47]. Subsequently, elements of a realist review were used to ask additional, targeted questions of the literature relating to how, why, and in what contexts certain study outcomes occurred within specific groups and environments [48,49].

### 2.1. Searching the Literature

**Figure A1:**
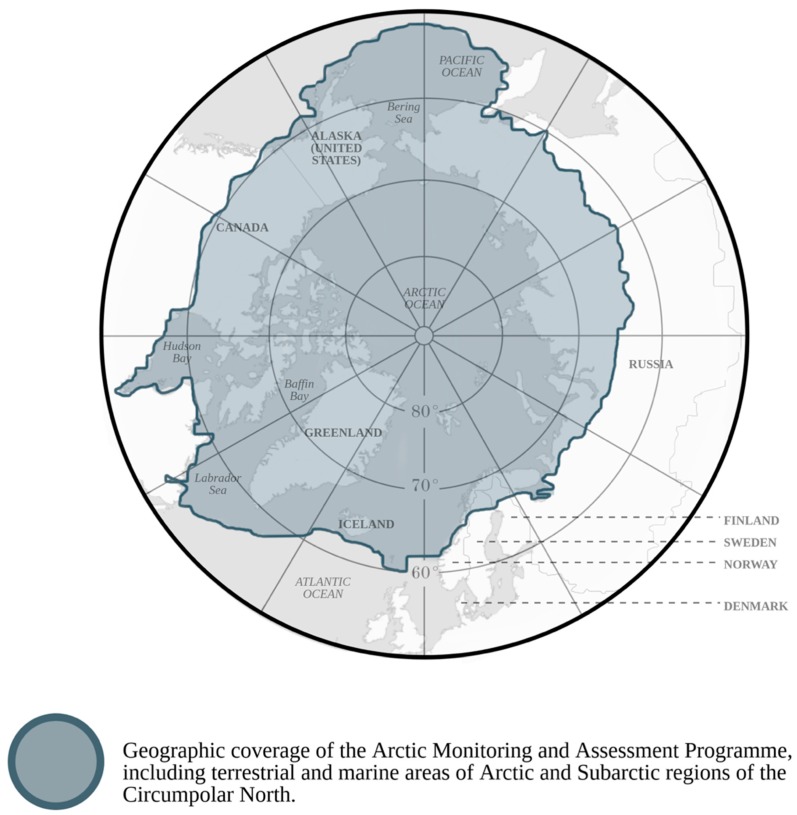
Map denoting the boundary area of Arctic and Subarctic regions of the Circumpolar North drawn from the geographic coverage defined by the Arctic Monitoring and Assessment Programme [170]. This boundary area includes terrestrial and marine areas of northernmost parts of eight Circumpolar countries (Canada, the Faroe Islands, Finland, Iceland, Norway, Sweden, Russia, and the United States) that were classified as Arctic or Subarctic climate regions under the Köppen classification system. This area extends as far south as 51.1°N to include areas north of the Aleutian chain, Hudson Bay, and parts of the North Atlantic Ocean, including the Labrador Sea.

Initial search terms were generated and refined through consultations with a research librarian to capture literature on integrated surveillance research and practice within Circumpolar Arctic and Subarctic regions. For the purposes of this systematic realist review, a combination of political, administrative, as well as geographic and climatic criteria were used to define “Arctic and Subarctic regions”. These regions were defined as the northernmost parts of eight Circumpolar countries (Canada, the Faroe Islands, Finland, Iceland, Norway, Sweden, Russia, and the United States) that were classified as Arctic or Subarctic climate regions under the Köppen classification system (Appendix A, Figure A1). The finalized search string (Table 1), was used to search MEDLINE^®^ and Web of Science™ databases in July 2015. Another search of these databases was conducted in June 2017 to capture literature published between August 2015 and December 2016. All searches were restricted by language (English), article type (articles, reviews, editorial materials, corrections), region (Arctic and Subarctic regions of Circumpolar countries), and date of publication (2005–2016). Searches were restricted to articles published after 2005 to capture articles published within the time period when human health began to take a more dominant role in Arctic environmental research [50]. A hand search of three key journals (*International Journal of Circumpolar Health; Arctic;* and *Environmental Health Perspectives*) was performed in June 2017 to assess the sensitivity of the initial database searches and identify any potentially relevant articles that were not captured in the database searches.

### 2.2. Selecting Studies

Article citations obtained through the database search were uploaded onto the Mendeley™ reference management tool (Mendeley Ltd., London, UK, v1.19.2), and subsequently exported to DistillerSR© software (Evidence Partners, Ottawa, ON, Canada, v2.12.0), to remove duplicates and facilitate screening. Two independent reviewers then conducted a two-stage screening process to select relevant articles. First, titles and abstracts of each article were screened based on a set of pre-determined inclusion and exclusion criteria (Table 2). All potentially relevant articles proceeded to the second stage of screening, where the full texts of these articles were reviewed using the same set of criteria. The level of agreement between the two independent reviewers was measured using Cohen’s Kappa (κ) for both stages of screening, with kappa values ≥ 0.80 indicating excellent agreement [51]. Conflicts between reviewers were discussed and reconciled regularly throughout the review process. 

### 2.3. Extracting, Analyzing, and Synthesizing Data from Relevant Articles

Extraction, analysis, and synthesis of data from relevant articles followed an iterative process [48]. Data extraction forms were created in DistillerSR© to gather descriptive information on general study characteristics, as well as information pertaining to the goal and objectives of this review (Appendix B, Table A1). Included articles were then uploaded onto NVivo© (QSR International, Burlington, MA, USA, v11), a qualitative data-management software, to facilitate a comprehensive thematic analysis of the inherent attributes and components of integrated environment and health surveillance systems in Circumpolar Arctic and Subarctic regions. An analytical framework for the identification of integrated surveillance attributes as described or recommended in the included articles was then developed (Appendix C, Table A2). This framework was informed by guidelines for public health surveillance attributes set by the European Centre for Disease Prevention and Control (ECDC) [52], the Centers for Disease Control and Prevention (CDC) [44], as well as literature on evaluating other forms of public health surveillance tools [45,46,53,54]. Enabling components of integrated surveillance systems were then identified through a process of inductive and deductive coding and thematic analysis, using a constant-comparative approach [55,56,57].

## 3. Results

**Figure 1:**
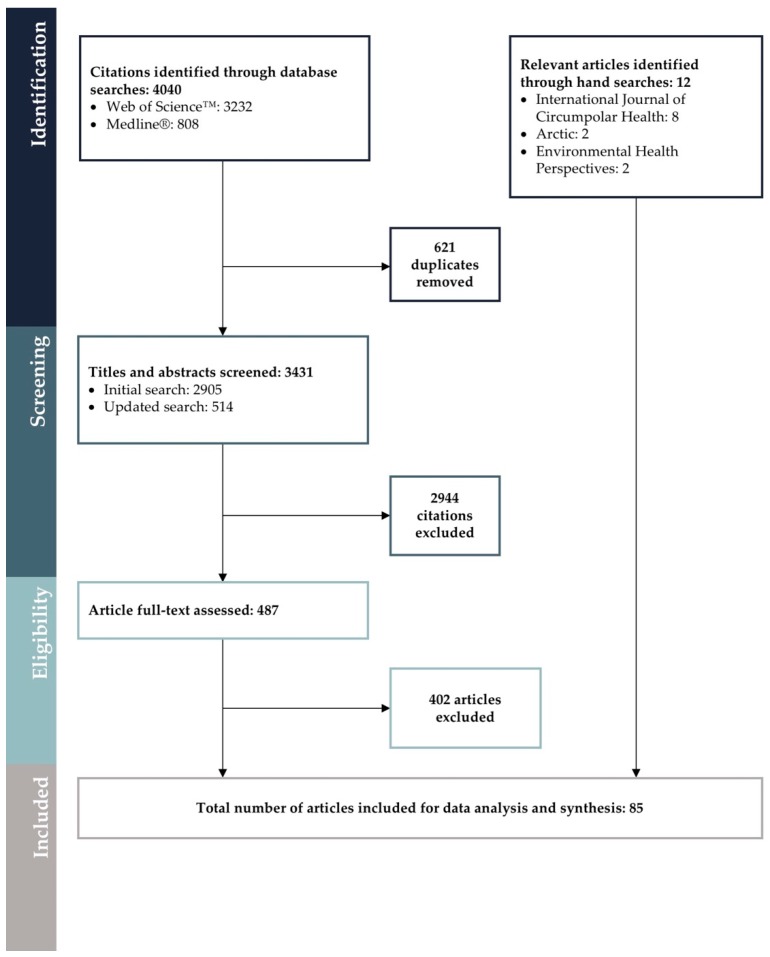
Diagram depicting the flow of identification, screening, and eligibility assessment of articles included in this review (*n* = 85). Rationale for exclusion of full-text articles is provided in Appendix D.

Database searches retrieved a total of 3431 unique citations, and 73 articles met all of the inclusion criteria. Hand-searching key journals identified 12 additional articles included for review that were not identified in the database searches. In total, 85 articles were included for data extraction, analysis, and synthesis (Figure 1; Appendix D, Table A3).

### 3.1. Descriptive Characteristics of Integrated Surveillance Systems

**Figure 2:**
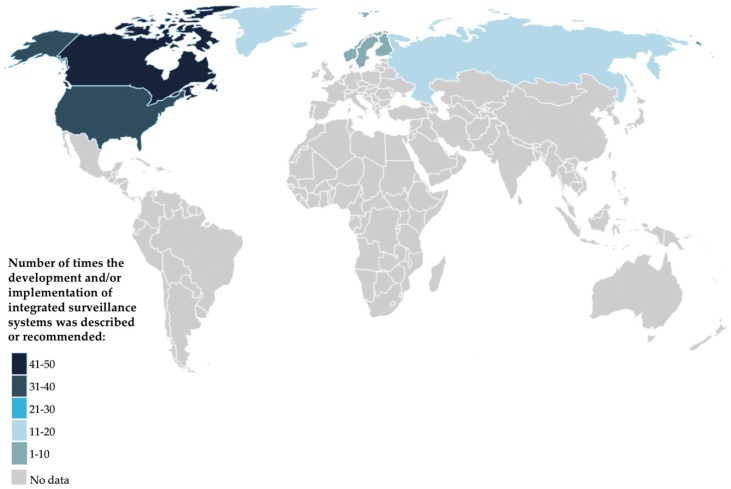
A map showing the Circumpolar countries where the literature described and/or recommended the development and/or implementation of integrated surveillance systems in Arctic and Subarctic regions (2005–2016), as distinct from the number of published articles per country. The darker the shade, the higher the relative proportions of mentions of integrated surveillance in that particular Circumpolar country.

The 85 included articles were categorized into one of two groups. The first group of articles (*n* = 55) informed, reviewed, and/or recommended integrated surveillance systems, wherein 58% were primary studies (*n* = 32) and 42% were secondary studies (*n* = 23). The second group of articles was comprised entirely of primary studies that focused on the development and/or implementation of integrated surveillance systems (*n* = 30). The majority of articles described integrated surveillance solely for research purposes, while very few described integrated surveillance for public health practice (*n* = 17; 20%). While most of the included articles described or recommended integrated surveillance within particular Arctic and Subarctic regions of Canada (*n* = 35; 41%), the United States (*n* = 29; 34%), Russia (*n* = 5; 6%), Iceland (*n* = 3; 4%), and Sweden (*n* = 1; 1%), 17 articles described or recommended integrated surveillance systems within multiple Arctic and Subarctic regions (20%) (Figure 2; Appendix E, Table A4). 

**Figure 3:**
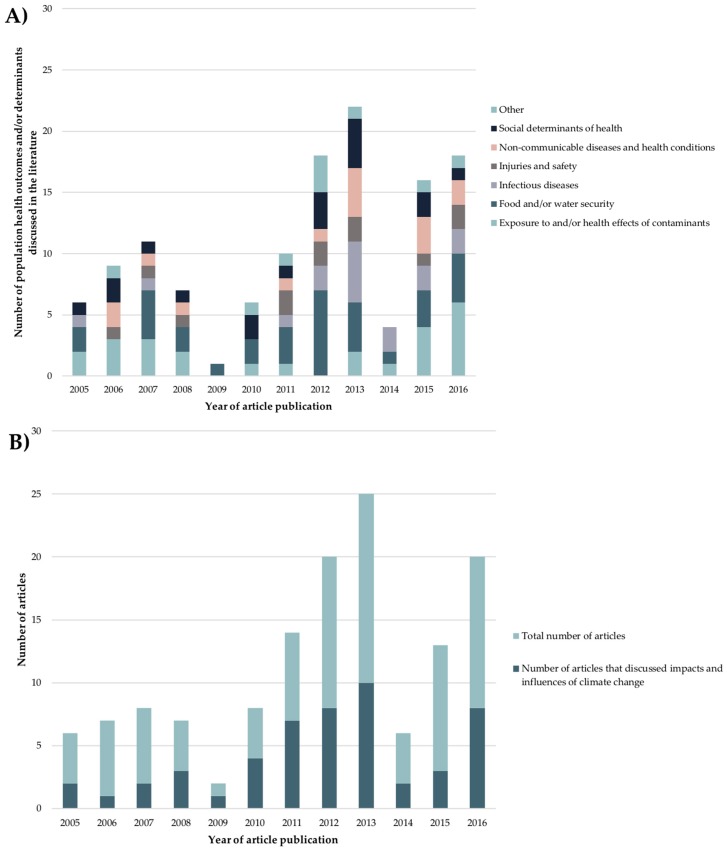
Timeline showing the year of article publication for literature on integrated surveillance for Circumpolar Arctic and Subarctic regions, stratified by (**A**) specific population health outcome or determinant, and (**B**) the number of articles discussing the impacts and influences of climate change on population health. Categories are not mutually exclusive.

Articles discussed integrated surveillance systems that were designed or recommended to monitor and enable responses to a wide range and diversity of environmental hazards, exposures, and/or conditions associated with population health outcomes and determinants. Several articles also discussed the need for and contributions of integrated surveillance to address impacts of climate change on these population health outcomes and determinants (Figure 3). The greatest numbers of distinct health population health outcomes and determinants were discussed in the literature on integrated surveillance in 2012 (*n* = 18), 2013 (*n* = 22), and 2016 (*n* = 18). In these same years, the greatest numbers of articles discussed impacts and influences of climate change on population health relative to the total numbers of articles published in those years. 

**Figure 4:**
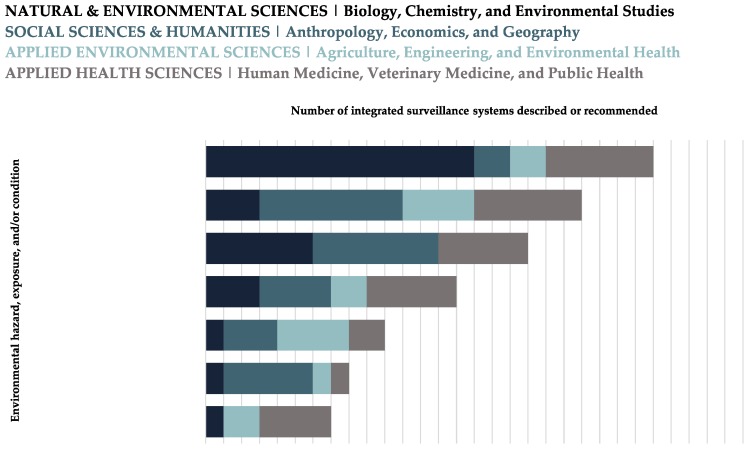
The main environmental hazards, exposures, and/or conditions included in integrated surveillance systems designed or recommended for Circumpolar Arctic and Subarctic regions, stratified by discipline (i.e., the first author’s primary discipline), as reported in the literature. The first author’s primary discipline was identified via the department they were affiliated with when the article was published. Affiliations were typically listed on the first page of each article, and any affiliations that were not listed were obtained via internet searches. Categories are not mutually exclusive.

Integrated surveillance systems were described from multiple disciplinary perspectives, with the greatest number of distinct disciplines contributing to literature on monitoring: environmental contaminants (e.g., [58,59,60]); meteorological change and variability (e.g., [61,62,63]); resource development (e.g., [64,65,66]); extreme weather events (e.g., [67,68,69]); and ice dynamics (e.g., [70,71,72]) (Figure 4). Most disciplinary areas described integrated surveillance for a wide range of environmental hazards, exposures, and/or conditions. 

**Figure 5:**
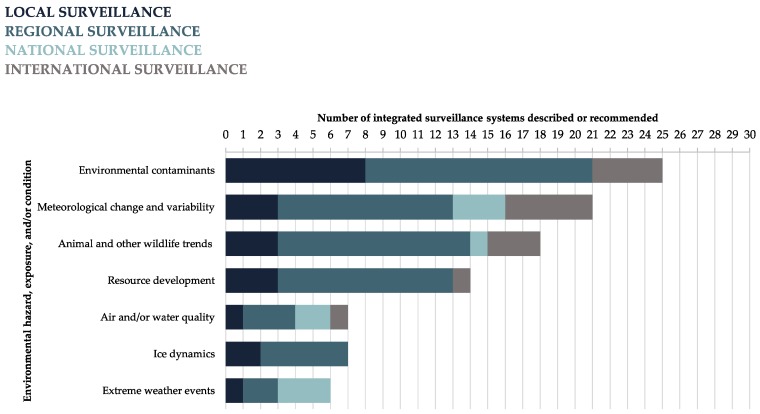
The main environmental hazards, exposures, and/or conditions included in integrated surveillance systems designed or recommended for Circumpolar Arctic and Subarctic regions, stratified by level of surveillance operation, as reported in the literature. Categories are not mutually exclusive.

Additionally, 44 (52%) of the integrated surveillance systems discussed in the included articles operated at a regional level, or within a specific province, state, or territory of a Circumpolar country. Of the remaining surveillance systems, 17 (20%) operated at a local level, eight (10%) at a national level, and 16 (19%) at an international level. Integrated surveillance systems were described across all levels of operation (i.e., local, regional, national, and international) for population health outcomes and determinants associated with air and water quality (e.g., [73,74,75,76]), wildlife trends and health (e.g., [76,77,78,79]), and meteorological change and variability (e.g., [62,80,81,82]) (Figure 5). Local and regional integrated surveillance systems were described or recommended for population health outcomes and determinants associated with every category of environmental hazards, exposures, and/or conditions identified in this body of literature.

Integrated surveillance systems identified in this review were described or recommended to serve a number of intended uses. One of the most commonly mentioned uses was to measure and assess the magnitude, scope, and/or distribution of the impacts of climatic and other environmental changes and variability on human health outcomes of importance (e.g., [83,84,85,86]). Integrated surveillance systems and data were also used to provide early warnings of changes in the environment that could pose potential risks to human health (e.g., [68,80,87]), and to guide public health resource allocation (e.g., [88,89]).

### 3.2. Attributes of Integrated Surveillance Systems

Using guidelines for public health surveillance attributes set by the ECDC, the CDC, and other relevant literature, ten attributes of integrated surveillance systems in the Arctic and Subarctic regions were identified within the included articles: acceptability (48%; e.g., [66,90,91]), data quality (48%; e.g., [86,92,93]), stability (47%; e.g., [94,95,96]), reliability (39%; e.g., [75,81,88]), relevance (38%; e.g., [89,97,98]), representativeness (36%; e.g., [74,99,100]), timeliness (34%; e.g., [89,91,101]), scalability (28%; e.g., [76,102,103]), flexibility (21%; e.g., [104,105,106]), and simplicity (12%; e.g., [58,73,90]) (Appendix C, Table A2). Surveillance attributes described and/or recommended in the literature were related to the purpose and intended uses of integrated surveillance systems and data, as well as to each system’s operational context. For instance, in articles that described integrated surveillance systems used for the identification of changes in human risk of exposure to environmental contaminants over time, considerations for attributes such as system stability and relevance were often reported (e.g., [103,107]). Stability was a particularly important attribute when a consistent, context-specific supply of surveillance information was needed to help address or explain environmental shifts that had slower, gradual effects on human health (e.g., [100,108]).

The nature of how certain surveillance attributes were described and interpreted within each article was also influenced by the needs of particular stakeholders that were involved in the development or implementation of integrated surveillance systems, and/or that used surveillance data. For example, some articles discussed considerations for flexibility in surveillance with regard to the need to seek input from stakeholders to guide the continuous improvement of a system (e.g., [58,84,96,106,109]). Other articles discussed considerations for flexibility when expanding a system to address similar surveillance needs in other contexts (e.g., [63]). Flexibility was also reported to be important when adjusting the types of communication strategies used to engage key stakeholders in prioritizing the goals for an integrated surveillance system (e.g., [60]).

Notably, interpretations of certain surveillance attributes also varied, based on the particular phase of integrated surveillance system development or implementation described in each article. For example, considerations for acceptability in articles that focused on earlier phases of integrated surveillance system development were sometimes described in relation to the identification and use of indicators and/or sentinel species for monitoring that would be most acceptable by stakeholders’ standards (e.g., [73,110,111]). In articles that described surveillance systems in later phases of implementation, considerations for acceptability were more often related to ensuring that communication, decisions, and actions based on surveillance information were acceptable within the population of interest (e.g., [70,84,85]).

The level and location of integrated surveillance system operation also influenced interpretations of certain surveillance attributes described in the included articles. Scalability, for instance, was in some cases discussed in relation to the horizontal application of surveillance systems, data, and/or tools across local or regional contexts (e.g., [66,84,90,99,101,112,113]). In other cases, scalability was related to a system’s potential for connectivity with national or international surveillance. These connections could facilitate the application and expansion of collective understandings of environmental changes and impacts on human health in a broader Circumpolar context (e.g., [70]).

### 3.3. Enabling Components of Integrated Surveillance Systems

**Figure 6:**
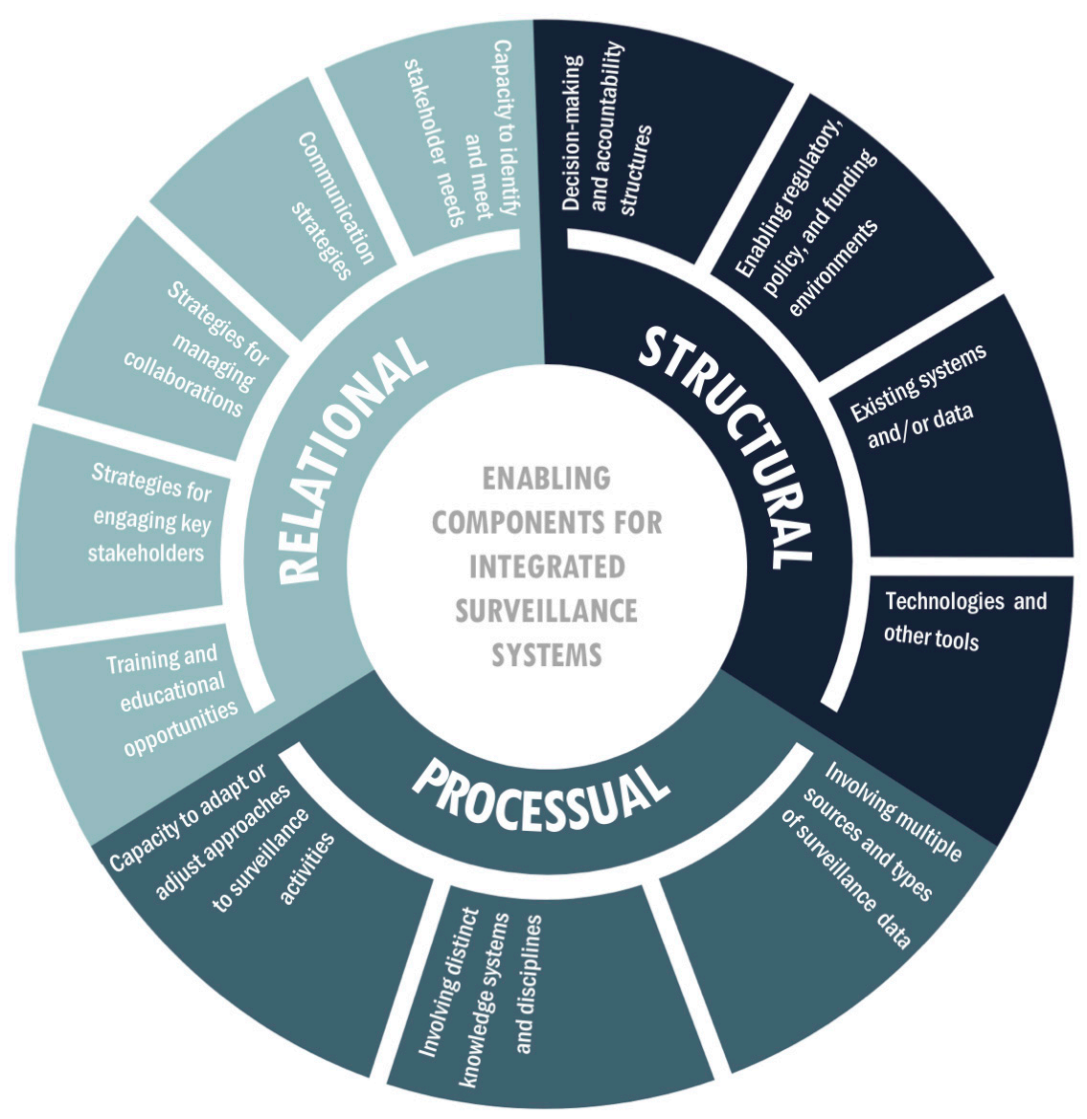
A visual display of the relationship between structural, relational, and processual components that enhanced and enabled attributes and activities of integrated environment and health surveillance systems in Circumpolar Arctic and Subarctic regions, as represented in the literature.

Articles described several components of integrated surveillance systems that contributed to enabling and/or enhancing the surveillance attributes and activities. In effect, these components helped a system achieve its intended goals and objectives for addressing environmental and human health concerns within particular socio-ecological contexts (Appendix F, Table A5). Integrated surveillance systems described in this body of literature were comprised of various numbers, types, and combinations of components in relation to the systems’ intended uses. These components were further categorized based on their contributions to structural, processual, or relational aspects of the development and implementation of integrated surveillance systems (Table 3; Figure 6).

#### 3.3.1. Structural

Components that enabled the logistic, organizational, and operational aspects of integrated surveillance system development and implementation were categorized as “structural”. For example, clearly defined decision-making and accountability structures were mentioned in some articles as components that could help to ensure integrated surveillance activities were responsive to population-specific needs and priorities (e.g., [108,135]). As noted by Germain [87], who used surveillance data to guide the development of early-warning systems for avalanches, establishing clear roles and responsibilities for public health and government officials helped hold those individuals accountable for their actions and contributions to enacting rapid responses to keep populations safe.

Funding structures were described as a component of integrated surveillance systems that were part of enabling regulatory and policy environments that could contribute to enhancing system stability. Stability, in this sense, was especially important when systems were designed to monitor and respond to longer-term climatic and environmental changes (e.g., [58,65,68,108,109,130]). Some articles also reported that securing long-term funding for integrated surveillance activities could help subsidize relatively high travel and equipment expenses in northern regions (e.g., [76,109]), and also enabled and enhanced more timely data collection processes (e.g., [63]).

Some articles described how existing environment and/or health surveillance systems or data were relatively inexpensive components that helped to efficiently establish the baseline health status of a population (e.g., [69,93,123,124,131,132]). From these baselines, public health priorities could be identified that were representative of that population (e.g., [90,113,121]). In this regard, several articles mentioned use of surveillance data from the Arctic Monitoring and Assessment Programme to examine trends in the health effects of environmental changes, and subsequently identify knowledge gaps for additional integrated surveillance efforts to fill (e.g., [60,63,90,91,102,103]). Additionally, Nilsson et al. [76] noted that many indicators for food and water security were already regularly monitored or surveyed in many Arctic countries. As such, the authors argued that the development of additional integrated surveillance systems for monitoring food and water security should involve efforts to obtain this existing information so as to avoid unnecessary exhaustion of time and resources. 

The literature described many integrated surveillance systems that used technologies such as remote-sensing, satellite imagery, and Global Positioning Systems that offered additional or enhanced surveillance capabilities, including communication of health risks (e.g., [68]), and weather forecasting (e.g., [78]). Laidler et al. [70], however, identified some challenges when using certain technologies in northern regions, due to extreme climatic and weather conditions. The authors further described how these technologies alone often did not provide sufficient context for using surveillance data to interpret and make predications at a local level.

#### 3.3.2. Processual

“Processual” components described in the included articles contributed to enabling and enhancing approaches for collecting, analyzing, managing, and interpreting surveillance data. For example, the capacity to adapt data collection and analysis activities was reported to help tailor integrated surveillance systems to serve local needs and regions of interest (e.g., [70]). Adaptive approaches to surveillance also helped address stakeholder concerns as they arose (e.g., [58]). Further, Nilsson et al. [76] noted that the capacity to adapt step-wise, iterative approaches for indicator identification and prioritization helped to generate a set of acceptable indicators that people would be willing to monitor.

With respect to surveillance data collection and interpretation, several studies described multiple methods of data collection and analysis to capture a wider range and depth of information on environmental and human health trends (e.g., [89,91,104]). Tremblay and colleagues used a combination of methods [89], who linked quantitative data from local weather stations and qualitative data from semi-structured interviews with Inuit community members about local-scale sea ice dynamics. In turn, these data produced more relevant information to support health-related climate change adaptation in Nunavik. Also, Driscoll and colleagues [84,85] used multiple modes of data collection (e.g., phone surveys, paper surveys, online tools) within one surveillance system in an effort to increase response rates and contribute to a more complete dataset.

Several distinct groups of stakeholders were reported to have varying degrees of involvement in the activities of collecting, analyzing, and interpreting surveillance data. These stakeholder groups included: government representatives from various departments and jurisdictional levels (e.g., [66,72,75,87,116]); health authorities (e.g., [67,92,113,121]); industry managers (e.g., [96,100,112,129]); researchers across distinct disciplines (e.g., [63,84,85]); and community members (e.g., [70,86,109,134]). Articles described how involving multiple stakeholder groups within integrated surveillance systems could help establish rigorous, transparent methods of addressing environmental and human health concerns that stakeholders were willing and able to support (e.g., [114,117]). Articles also described how engaging with and drawing upon knowledge systems of Indigenous and non-Indigenous researchers, governments, and communities could help strengthen surveillance capacity to gain a more representative, holistic picture of the interconnected impacts of climate and environmental changes on human wellbeing (e.g., [70,106,122,141]). For instance, Brook et al. [109] described how Indigenous knowledge of local environmental conditions in the Sahtu Settlement Area (Northwest Territories, Yellowknife, NT, Canada) was an essential component of a surveillance system designed for detecting and tracking wildlife health trends that were relevant to the local population.

While many articles discussed how the complementary nature of Western and Indigenous knowledge systems contributed to understandings of complex environment-health phenomena (e.g., [105,108,109]), others recognized differences inherent in distinct knowledge systems that, in turn, led to new understandings. As explained by Pennesi et al. [62], Inuit knowledge of weather conditions in Iqaluit, Nunavut sometimes conflicted with forecasting data from weather stations. However, the authors found that these distinct sources of weather information were used for different surveillance purposes, depending on whether the information was being used for decision-making in the short- or long-term.

#### 3.3.3. Relational

“Relational” components, or the interpersonal components of integrated surveillance development and implementation, contributed to building and maintaining connections between stakeholders and often enabled surveillance systems to be more responsive to stakeholder needs and priorities. Indeed, several articles noted that strategies for bringing together key stakeholders, such as government agencies, industries, academic researchers, co-management bodies, and Indigenous rights-holders, could help to achieve mutually beneficial outcomes for all those involved (e.g., [58,70,76,84,85,89,106,109,134]). Articles that described surveillance systems involving multiple stakeholders also often emphasized the importance of having the capacity to facilitate collaboration between those stakeholders to support surveillance design, data collection, and its use. Collaboration strategies were further described as a means of generating trust between stakeholders (e.g., [106]), identifying objectives, and guiding regular evaluation (e.g., [84,134]), which was also reported to help democratize surveillance activities by distributing tasks, duties, and resources amongst many stakeholders (e.g., [109]). Moreover, as noted by Burger et al. [96], the capacity to facilitate meaningful, consistent dialogue within and between multiple stakeholders helped to identify potential gaps in surveillance data collection that were not always possible to see from a single perspective. Through the identification of these gaps, stakeholders were better able to ensure and enhance surveillance data completeness.

Consultations were described as another relational component that helped facilitate connections with key stakeholders, which, in turn, could enable opportunities for equitable decision-making processes and knowledge exchange (e.g., [70,96]). Driscoll et al. [84,85] explained how consultations with local experts in Alaskan communities led to more deliberate development processes for surveillance indicators and tools that were particularly useful in regions of Alaska with few secondary data-sources. In other articles, consultations with international experts helped identify comparable surveillance indicators to monitor environmental changes and associated health impacts in systems implemented across multiple northern regions (e.g., [76]).

The literature also described surveillance systems that included the capacity to engage specific stakeholders, as well as beneficiaries of surveillance data, throughout the development and implementation of a system. Effective stakeholder engagement strategies could enhance the overall acceptability of a surveillance system (e.g., [84,89,109,126,134]), and also ensure usefulness of surveillance data (e.g., [70,84,89]). In particular, engaging community stakeholders was emphasized in many articles that used community-based approaches to identify locally relevant environment and health priorities and associated actions prior to the development of a surveillance system, which could also contribute to local acceptance of a system (e.g., [70,84,85,90]). Further, efforts to initiate and sustain community engagement could contribute to the following priorities of particular surveillance systems: enabling more timely collection and application of surveillance data (e.g., [89]); generating activism and advocacy surrounding environmental issues (e.g., [86]); offering opportunities to foster youth leadership and cross-generational learning (e.g., [134]); and supporting continuity of surveillance beyond the end of a project or funding cycle (e.g., [109]). 

Several studies described how training and educational opportunities could serve as components that helped build capacity among academic researchers, funders, and governments to develop the necessary skills and knowledge required for developing and implementing integrated surveillance in northern contexts (e.g., [68,121,123]). Training opportunities were reported to help create opportunities for early-career researchers (e.g., [86]), and establish frameworks from which to pursue larger public health strategies (e.g., [109]). These types of opportunities could also enable the generation of higher-quality data. For example, Brook et al. [109] trained local Wildlife Health Monitors to collect and record data, which served to ensure local validity of data and also contributed to enhancing local acceptability of decisions made based on those data. 

## 4. Discussion

Findings from this review demonstrated that literature on integrated environment and health surveillance in the North for both research and practice spanned multiple Circumpolar countries [34,41]. Examining the nature of integrated surveillance as described in the literature provided insights into the ways in which various types of surveillance systems and data contributed to improving public health capacity for addressing direct and indirect impacts of increasing climate change on population health outcomes and determinants in northern regions [122,143]. Outside of this literature, several advantages of integrating meteorological, ecological, and epidemiological surveillance datasets have been described as useful for identifying trends and geographical exposure areas, for issues such as climate-sensitive infectious diseases [144], heatwaves [145], and extreme weather events [33]. Analyses and interpretations based on these integrated surveillance datasets can provide information that is useful for climate change adaptation. This information can help to direct resources toward communities determined to be most at risk, providing evidence-based support for developing and funding public health services, as well as informing the distribution of relevant public health information [5]. Taking advantage of the opportunities brought about by integrated surveillance is particularly important for northern populations that are experiencing some of the most rapid climate change and associated environmental shifts globally [3,9]. 

The large number of articles retrieved from North American regions, however, illustrated a relatively strong geographic concentration of integrated environment and health surveillance research that may not adequately represent surveillance priorities of other Circumpolar regions. This finding could be influenced by the English and French language restriction, as well as the increasing calls for integrated surveillance from North American governments and funding bodies over the past several years [8,146]. Circumpolar countries are highly diverse geographically, ecologically, and socioeconomically, and so the types and levels of risks from climate change experienced across these countries vary between populations, depending on vulnerability and exposure to hazards, adaptive capacity, and risk perceptions [5]. Priorities for climate change adaptation between and within these countries will necessarily be context-specific [11]. Developing integrated surveillance systems that are aligned with population- and region-specific priorities will thus require careful consideration for the needs of potential users and beneficiaries of surveillance data [147]. In this light, it is also important to consider the contextual, temporal, and spatial aspects of pathways through which global climate change impacts population health outcomes and determinants [148]. 

Indeed, pathways through which climate change impacts population health tend to be complex, indirect, diffuse, and/or delayed, and often interact with many other socio-ecological factors that influence human health [144,149]. The complexity of climate change-related impacts on human health will also determine adaptation options and barriers in particular regions [150]. Integrated surveillance systems can serve as tools for improving basic understandings of relationships between climatic and environmental conditions and human health in the context of the socio-ecological processes within which these relationships are generated [143]. In effect, understandings afforded by the analysis and interpretation of integrated surveillance data can help to identify potential gaps in climate change adaptation research and practice, that are due in part to the complexity of socio-ecological processes [144]. Collecting these data consistently over time can provide a means of tracking progress in minimizing these gaps [151].

A variety of disciplinary lenses were used to guide research on integrated surveillance development and implementation across multiple levels of operation, and helped contribute to both individual and collective goals for enhancing and sustaining population health within drastically changing northern environments [152,153]. As climate change continues to exacerbate existing population health issues and places additional stress on public health resources [143], there is a pressing need for adaptive actions that involve coordinated, multi-level approaches. These approaches will rely upon input from stakeholders across disciplinary and jurisdictional boundaries to help make decisions regarding resource allocation for the monitoring and surveillance of climate-sensitive health outcomes [143,144,154]. In this light, findings from this review reinforced the important contributions of communities, academic researchers, governments, and other stakeholders—spanning multiple disciplines and sectors—in designing integrated surveillance systems that served particular uses within specific contexts and at various levels. Considered alongside the collective call for integrated surveillance in the North [7,27,28], these findings demonstrated how integrated surveillance systems were designed to serve a wide range and diversity of uses, where each provided important types of data and information for guiding timely, targeted, evidence-based public health responses in specific locations in support of broader goals for adapting to change [122,151]. 

As evidenced by the surveillance systems described in the included articles, addressing large, complex challenges at the intersection of environmental and human health in the Circumpolar Arctic and Subarctic regions required surveillance systems that were comprised of various structural, processual, and relational components. Considering the connections and interactions between these components could serve to enable and enhance key surveillance attributes and activities. While some of the components identified here have also been discussed in relation to other forms of public health surveillance [42,53,122], findings from this review can be used to inform decisions surrounding if and when certain surveillance components, and/or combinations of components, should be emphasized or introduced to guide more appropriate and effective climate change adaptation strategies. For example, the Intergovernmental Panel on Climate Change recommended improved communication, education, and training as some of the key focal areas of adaptation strategies for dealing with climate-related risks in northern regions, particularly for risks related to human health and wellbeing [5]. Building and enhancing existing integrated surveillance systems that prioritize relational components, such as communication and outreach, could thus offer mutual benefits for climate change adaptation in the North. 

This review aimed to provide a holistic picture of how certain components were linked to surveillance attributes and activities. In turn, findings offered insights into the operations and management of surveillance systems that served particular uses for addressing environmental and human health concerns. Identification of surveillance attributes and enabling components was limited by the level of detail articles used to describe or recommend integrated surveillance systems. Further challenges arose when attempting to apply broad definitions of public health surveillance attributes across a wide range and diversity of systems, all serving distinct purposes and stakeholder priorities. As noted by Auer et al. [54] in their review of injury surveillance system evaluations, there is a high degree of variability in selecting surveillance attributes that would be most important for an individual surveillance system, as well as differences in how those attributes are defined and interpreted between surveillance systems. Understanding how structural, processual, and relational components can work together to enhance and enable key integrated surveillance attributes and activities in the context of climate change can help researchers and practitioners plan and evaluate integrated surveillance systems that are more responsive to public health concerns within rapidly shifting northern environments [155,156]. For instance, the acceptability of an integrated surveillance system could be evaluated based on the extent to which community and cultural values are included in the collection and communication of surveillance information to inform climate change adaptation strategies [157,158]. However, there is a need for further study into methods for selecting the most relevant attributes to assess, monitor, and evaluate in order to provide relevant recommendations for improving the ways in which integrated surveillance systems can contribute to climate change adaptation within variable socio-ecological contexts. Evaluation criteria for improving the performance and efficiency of these systems must be flexible enough to allow for these variations [46]. Further, when developing and using these criteria, evaluators must also anticipate the potential for increasing climate change to create additional challenges for population health and place additional demands on integrated surveillance capacity.

This review focused solely on integrated surveillance as represented in the academic literature, and articles mainly focused on surveillance for research purposes. Without considering government reports and other grey literature, this review likely did not capture literature on other forms of surveillance that would be useful for developing deeper understandings of the ways in which certain surveillance components enable and enhance integrated surveillance attributes and activities for public health practice, or for identifying additional components that were not discussed in these articles. For example, many Indigenous communities in the North also often practice local, informal monitoring that is not always well-represented in the academic literature [159,160]. These forms of monitoring could offer different types of tools and insights for enhancing integrated surveillance in the context of climate change [43]. Ultimately, including these perspectives in developing, implementing, and evaluating integrated surveillance systems is essential for optimizing the utility and relevance of surveillance information and data for decision-making and resource allocation within public health policy and programming that is linked with community-specific goals for climate change adaptation [122]. Moreover, many Indigenous communities in the North and globally have, historically, lacked control and access to their own health data [161,162]. In this light, future studies focusing on integrated surveillance research and practice within Indigenous homelands in northern regions should prioritize engagement with Indigenous communities and representational organizations as rights-holders in setting surveillance priorities and determining how data and information about people and the environment is collected, stored, used, and shared for meaningful climate change adaptation [163]. Indeed, community engagement is critical for understanding how climate change will disproportionately and differentially impact northern communities. Thus, adaptations must be rooted in local customs, values, and decision-making process if they are to be successful, building on community-specific ways of knowing, monitoring, and adapting to local environmental conditions [164].

Finally, these findings revealed a relative lack of discussion in the academic literature regarding how to effectively monitor and respond to the impacts of climate change and associated environmental shifts on the some of the more intangible dimensions of population health and wellbeing [165,166,167]. Emotional wellbeing [24,25], attachment to place [14], as well as cultural and spiritual aspects of health, are among the many intangible dimensions of health and wellbeing that are increasingly recognized as priorities for Indigenous and non-Indigenous communities, in the North and globally [168,169]. As surveillance systems and data can only be fully understood in the context of specific health outcomes [35], there is a need for research that connects these intangible dimensions of wellbeing with priorities and corresponding approaches for using integrated surveillance data and information to guide more comprehensive climate change adaptation strategies. Effective adaptation to the impacts of climate change on human health must take into consideration the cultural values that are important for community and individual wellbeing, as well as the many, interconnected impacts climate change can have on population health outcomes that are not as easily measured or quantified [5]. For example, increasing climate change is associated with elevated risks to mental health and wellbeing through factors that interact to produce loss of personal resources, lead to widespread destruction and upheaval, and that place additional pressure on public health-related resources [170]. Without a comprehensive understanding of how climate change interacts with other socio-ecological factors to impact all dimensions of wellbeing, adaptations to the impacts of climate change on the health of populations will be inadequate and/or incomplete. Further developing this understanding is particularly important for populations whose health and wellbeing is intimately connected to the environment and who are simultaneously experiencing drastic and rapid climate change impacts, both in the Circumpolar North and globally.

## 5. Conclusions

By examining and synthesizing literature on integrated surveillance research and practice in northern regions, this review provided information to improve understandings of how environment and health data can be integrated and interpreted to inform evidence-based public health strategies, guidelines, primary healthcare services, and policy development in support of climate change adaptation. This review established an initial conceptual framework for understanding the range, diversity, and enabling components of integrated surveillance systems. Within this initial framework exist important opportunities for future research that can contribute to characterizing the types and contributions of integrated surveillance systems to public health practice in the North. Further, there exist many potential uses for integrated surveillance data in identifying public health priorities and enhancing public health capacity to adapt to rapid, unprecedented climate change and associated environmental shifts. 

Beyond the characterization of structural, processual, and relational integrated surveillance components, these findings demonstrated the importance of asking additional questions pertaining to how combinations of components within a surveillance system could enable and enhance key surveillance attributes and activities. A more thorough understanding of the components that comprise integrated surveillance attributes can inform how, and in what contexts, certain attributes should be selected and evaluated, and can also help improve surveillance activities and capabilities in relation to intended goals and objectives for addressing environmental and human health concerns within a given population. These questions become especially important as climate change continues to create new, complex challenges in terms of public health preparedness for, and responses to, the potential impacts on population health. Understanding of how integrated surveillance systems are designed to operate and serve specific, yet not mutually exclusive end-uses for improving population health in the context of climate change can help communities, researchers, governments, and other stakeholders in decision-making, resource allocation, and continuous improvement of integrated surveillance research and practice. This, in turn, can guide the development, operation, and evaluation of appropriate surveillance systems that link public health priorities with climate change adaptation.

## Figures and Tables

**Table 1:** Finalized search strings for Web of Science™ and MEDLINE^®^ aggregator databases to identify articles related to integrated surveillance strategies in Arctic and Subarctic regions of the Circumpolar North that involved considerations for the natural environment, human health, and surveillance (2005–2016).

	Web of Science™	MEDLINE^®^
**Natural environment terms ^1^**	((climat * NEAR/2 (change or variabl * or extreme)) or global warm * or ice or disaster* or fire * or cyclone * or storm * or flood * or drought * or rain or snow or (tidal NEAR/2 wave *) or tornado * or (food NEAR/2 (suppl * or safe * or security or quality)) or (water NEAR/2 (suppl * or fresh or drink * or security or quality or pollut *)) or weather or (extreme NEAR/2 (cold or heat)) or (air NEAR/2 (quality or pollut *)) or humidity or temperature * or wind * or “ultraviolet rays” or (environment * NEAR/2 (monitor * or medicine or health or pollut * or exposure *)))	((climat * adj2 (change or variabl * or extreme)) or global warm * or ice or disaster * or fire * or cyclone * or storm * or flood * or drought * or rain or snow or (tidal adj2 wave *) or tornado * or (food adj2 (suppl * or safe * or security or quality)) or (water adj2 (suppl * or fresh or drink * or security or quality or pollut *)) or weather or (extreme adj2 (cold or heat)) or (air adj2 (quality or pollut *)) or humidity or temperature * or wind * or ultraviolet rays or (environment * adj2 (monitor * or medicine or health or pollut * or exposure*))).tw.
**Surveillance terms**	((ecological * NEAR/3 monitor *) or (disease NEAR/2 notification) or ((surveillance or monitor * or track * or assess *) NEAR/3 (population or health* or environment *)) or ((prevent * or warn * or prepar * or surveillance or monitor * or track * or assess * or detect *) NEAR/3 (sentinel or health *)) or ((prevent * or warn * or prepar * or surveillance or monitor * or track * or assess * or detect * or adapt *) NEAR/3 system *) or (strategy * NEAR/3 (climat * or environment * or adapt *)))	((ecological * adj3 monitor *) or (disease adj2 notification) or ((surveillance or monitor * or track * or assess *) adj3 (population or health * or environment *)) or ((prevent * or warn * or prepar * or surveillance or monitor * or track * or assess * or detect *) adj3 (sentinel or health *)) or ((prevent * or warn * or prepar * or surveillance or monitor * or track * or assess * or detect * or adapt *) adj3 system *) or (strategy * adj3 (climat * or environment * or adapt *))).tw.
**Human health terms**	(health or wellbeing OR safe * or injur * or illness * or disease * or infect * or “frost bite *” or burn * or wound *)	(health or wellbeing OR safe * or injur * or illness * or disease * or infect * or frost bite * or burn * or wound *).tw.
**Geographic focus**	(Circumpolar or polar or “Arctic Canada” or Canada or Alberta or “British Columbia” or “New Brunswick” or Manitoba or “Newfoundland and Labrador” or “Northwest Territories” or “Nova Scotia” or Nunavut or “Prince Edward Island” or Ontario or Quebec or Saskatchewan or Yukon or Nunavik or Nunatsiavut or Inuvialuit or Norway or Svalbard or Greenland or Denmark or Alaska or “United States” or Russia or Sweden or Finland or Iceland or Scandinavia or “Nordic countr *” or Arctic or North *)	(Circumpolar or polar or Arctic Canada or Canada or Alberta or British Columbia or Manitoba or Newfoundland and Labrador or Northwest Territories or Nunavut or Ontario or Quebec or Saskatchewan or Yukon or Nunavik or Nunatsiavut or Inuvialuit or Norway or Svalbard or Greenland or Alaska or Russia or Sweden or Finland or Iceland or Scandinavia or Nordic countr * or Arctic or North *).tw.

^1^ Truncation symbols (*) were used at the end of a search term, or part of a search term, to broaden the searches by retrieving unlimited suffix variations. The proximity operators “NEAR/x” and “ADJx” were used within Web of Science™ and MEDLINE^®^ database searches, respectively, to retrieve records where the terms joined by the operator were within a specified number (x) of words of each other. The “.tw.” operator used in MEDLINE^®^ database searches indicates a free text search specifically within the title and abstract fields to search for keywords.

**Table 2:** Inclusion and exclusion criteria that were used in first-stage title and abstract screening and the second-stage full-text review to identify articles about integrated environment and health surveillance systems in Arctic and Subarctic regions of the Circumpolar North between 2005 and 2016.

	Inclusion Criteria	Exclusion Criteria
**Publication information**	Article was published in English or French	Not published in English or French
	Article was published between 2005 and 2016	Article was published before 2005 or after 2016
	Primary or secondary study was published in a journal article	Theses, conference proceedings, reports, commentaries, etc.
**Article context**	Main site/focus and implications of the article were within “Arctic and/or Subarctic regions,” referring to High Arctic, Low Arctic, and Subarctic geographic areas in Circumpolar countries (Canada, the Faroe Islands, Finland, Greenland, Iceland, Norway, Russia, Sweden, and the United States) with an Arctic or Subarctic Köppen climate classification	Main site/focus and implications of the article were outside Arctic or Subarctic regions of Circumpolar countries
**Article focus**	Article involved a biophysical environment-related change(s)/exposure(s)/issue(s)	Article involved an environmental change/exposure/issue that related to the built environment or “cultural landscape” created by humans
	Article included one or more outcome(s), condition(s), illness(es), disease(s), status(es), indicator(s), or determinant(s) related to the health and/or wellness of humans	Article did not involve any human health outcome(s), condition(s), illness(es), disease(s), status(es), indicator(s), or determinant(s)
	Article focused on surveillance, defined as the continuous, systematic collection, analysis, and interpretation of environment and health-related data needed for the planning, implementation, and evaluation of public health research and/or practice, integrated with the dissemination of these data to end-users [35]	Article did not focus on the development, implementation, use, or evaluation of surveillance strategies, systems, or research

**Table 3:** Attributes and enabling components of integrated surveillance systems in Circumpolar Arctic and Subarctic regions as described in the literature, comprised of articles that informed, reviewed, and/or recommended integrated surveillance, and articles that described the development, implementation, and/or application of integrated surveillance. Articles are sorted in descending order, based on the number of components described.

Articles informing, reviewing, and/or recommending integrated surveillance systems																								
	Acceptability	Data quality	Flexibility	Relevance	Reliability	Representativeness	Scalability	Simplicity	Stability or sustainability	Timeliness	Number of attributes described	Decision-making and accountability structures	Existing surveillance systems and/or data	Policy, regulatory, and funding	Technologies and tools	Capacity to adapt surveillance activities	Involving distinct knowledge systems	Multiple sources/types of data collection	Capacity to meet stakeholder needs	Communication strategies	Strategies for managing collaborations	Strategies for engaging individual stakeholders	Training and educational opportunities	Number of components described
Young et al. [91]											7													9
Berner et al. [90]											8													8
Donaldson et al. [60]											6													7
Ford et al. [79]											2													6
Parkinson [63]											4													6
Banfield and Jardine [66]											3													5
Amstislavski et al. [99]											6													5
Austin et al. [68]											0													5
Kwiatkowski [114]											2													5
Tsuji et al. [64]											3													5
Martin et al. [115]											4													5
Burger et al. [96]											4													4
Donaldson et al. [95]											6													4
Furgal and Seguin [72]											5													4
Gunnarsdóttir et al. [116]											3													4
Hueffer et al. [61]											4													4
McClymont Peace and Myers [105]											4													4
Metcalf and Robards [117]											2													4
Natalia [118]											1													4
Pearce et al. [119]											2													4
Smith et al. [120]											3													4
Wernham [65]											3													4
Brubaker et al. [121]											0													3
Noble and Bronson [112]											2													3
Parkinson and Butler [82]											3													3
Van Oostdam et al. [101]											4													3
Bronson and Noble [122]											4													2
Abass et al. [94]											2													2
Bhatia and Wernham [104]											3													2
Brubaker et al. [113]											1													2
Byrne et al. [93]											3													2
Dubé et al. [81]											3													2
Ford et al. [123]											1													2
Ford et al. [71]											3													2
Gadamus [78]											2													2
Krzyzanowski [74]											3													2
Lepak et al. [98]											2													2
Moiseenko et al. [124]											1													2
Pearce et al. [125]											2													2
Pennesi et al. [62]											2													2
Tomaselli et al. [77]											3													2
Bond et al. [83]											2													1
Ford et al. [126]											4													1
Gibson et al. [102]											3													1
Harley et al. [127]											1													1
Hori et al. [69]											2													1
Lynn et al. [128]											1													1
Noble and Bronson [129]											1													1
Provencher et al. [97]											5													1
Andrachuk and Smit [130]											1													0
Ding et al. [131]											2													0
Kirk et al. [111]											2													0
Konkel [132]											0													0
Kraemer et al. [59]											3													0
Rosa et al. [133]											3													0
**Articles describing the development and/or implementation of integrated surveillance systems**																								
	**Acceptability**	**Data quality**	**Flexibility**	**Relevance**	**Reliability**	**Representativeness**	**Scalability**	**Simplicity**	**Stability or sustainability**	**Timeliness**	**Number of attributes described**	**Decision-making and accountability structures**	**Existing surveillance systems and/or data**	**Policy, regulatory, and funding**	**Technologies and tools**	**Capacity to adapt surveillance activities**	**Involving distinct knowledge systems**	**Multiple sources/types of data collection**	**Capacity to meet stakeholder needs**	**Communication strategies**	**Strategies for managing collaborations**	**Strategies for engaging individual stakeholders**	**Training and educational opportunities**	**Number of components described**
Laidler et al. [70]											9													10
Tremblay et al. [89]											5													10
Brook et al. [109]											4													9
Wesche et al. [134]											5													8
Ford et al. [108]											3													8
Germain [87]											6													7
Vlasova and Volkov [100]											7													7
Driscoll et al. [84]											7													6
Fall [135]											4													6
Berkes et al. [106]											6													6
Gunnarsdóttir et al. [75]											4													6
Burger [58]											4													5
Larrat et al. [92]											8													5
Miller et al. [86]											4													5
Driscoll et al. [85]											8													4
Dudarev et al. [136]											4													4
Nilsson et al. [76]											6													3
Pacyna et al. [103]											6													3
Burger and Gochfeld [107]											5													3
Dagsson-Waldhauserova et al. [137]											4													3
Pardhan-Ali et al. [138]											3													2
Huntington et al. [139]											1													2
Khalil et al. [80]											3													2
Skandfer et al. [140]											3													2
Dunlap et al. [110]											5													1
Bruden et al. [88]											4													1
Burger et al. [141]											5													1
Ludwicki et al. [142]											4													1
Montrose et al. [73]											2													1
Do et al. [67]											4													0

## Appendix B

**Table A1:** Coding form for qualitative analysis and synthesis of the 85 included articles that described integrated environment and health surveillance in Circumpolar Arctic and Subarctic regions.

Data Extraction Categories	Questions
Article information	Title
	First author
	Year
	Journal
Geographic and methodological information	Study site, scope, and physical context—community, state/territory/province, country
	Population/community characteristics and socio-cultural context
	Methodology/study design
	Other methods-related notes
	Main purpose
Environmental considerations	What are the considerations/implications for the natural environment?
Human health considerations	What are the considerations/implications for human health?
Attributes and components of integrated surveillance systems	What type of surveillance is being informed/developed/used—active, passive, sentinel?
	Is the article informing, developing, or using integrated surveillance?
	Is “surveillance” explicitly described in the article?
	Describe any attributes related to integrated surveillanceDescribe any enabling components related to integrated surveillance
	How do the components described relate to surveillance attributes and enable surveillance activities?
Recommendations and implications	What are the recommendations?
	How do recommendations inform integrated surveillance?
	Lessons that can be applied to future integrated environment and health surveillance work?
	Describe any useful introductory or background material provided
Other	Weaknesses/limitations—what was not included or discussed in this article that would be useful to know?
	How will this article be used in the review?
	How does this article relate to other articles in the review?

## Appendix C

**Table A2:** Inherent attributes of integrated surveillance systems in Circumpolar Arctic and Subarctic regions described or recommended by the literature.

Attribute	Definition	Example
Acceptability	Willingness of interested stakeholders and/or end-users to participate and use the system	In the case of a community-based surveillance system described by Driscoll et al. [84], environmental exposures selected for surveillance had to be of sufficient priority to community members so that they were willing to contribute to the collection of primary data on those exposures.
Data quality	Completeness and validity of data, and processes of data acquisition	Dudarev et al. [136] sought to gather data on infectious and parasitic diseases in Arctic regions of Russia and Siberia and emphasized that data needed to be complete to enable accurate comparisons between locations and levels (i.e., regional and national levels).
Flexibility	Ability of system to adapt to changing needs or operating conditions with little additional time, personnel, or funding	In response to logistical and communication challenges identified by hunters, graduate students, and biologists in a community-based wildlife health monitoring program, Brook et al. [109] adapted their sample collection protocol and training of hunters.
Relevance	Ability of system to meet its intended purpose, as well as its practicality and affordability to operate	Pacyna et al. [103] used results from monitoring human exposure to persistent organic pollutants to inform strategies and policies on emission reduction in Arctic regions.
Reliability	Confidence in the reliability of surveillance data and information; encompasses sensitivity and positive predictive value	Nilsson et al. [76] used measures of seroprevalence of food-borne diseases in humans to identify populations at risk, as this was described as an indicator of high predictive value.
Representativeness	Ability of system to accurately describe the occurrence of an environment- or human health-related event over time, and describe its temporal and spatial distribution	In regular toxicological monitoring of wildlife subsistence hunts, Bond et al. [83] obtained samples from consumptive harvests to ensure results were representative of what communities were actually eating.
Scalability	Ability of surveillance systems and data to connect within and between levels and contexts of operation in Northern populations and environments	Laidler et al. [70] explained how the potential for connectivity between local surveillance systems and national or international datasets could help decision-makers identify and understand patterns and trends in environment and human health issues.
Simplicity	Considerations for ease of system design and operation	Collection of syndromic health data from trained stakeholders representing communities of interest, or sentinel communities, was reported by Driscoll et al. [84] to help bypass the need for clinical diagnoses, thereby making community-based surveillance systems simpler to operate.
Stability or Sustainability	Ability of system to continuously and consistently serve its intended purpose over time, and/or collect environment and health data to produce sufficient information for explaining and comparing dynamic, complex environment-health interactions	Dunlap et al. [110] used long term monitoring of lipid profiles of sled dogs, a sentinel species, to observe the synergies among threats to the subsistence lifestyles of Northern communities over time.
Timeliness	Ability of system to generate up-to-date information as needed, considered in terms of availability of information to inform public health responses	As recommended by Bronson and Noble [122], surveillance of environmental impacts of resource development should focus on monitoring determinants of human health to provide early warnings of the actual outcomes time lags in identifying climate-sensitive health outcomes.

Note: Definitions are based on guidelines for public health surveillance attributes set by the European Centre for Disease Control, the Centers for Disease Control, as well as other relevant literature on evaluating other forms of public health surveillance systems [44,45,46,52,53,54]. Corresponding examples of each attribute are provided, as identified in the 85 articles included for review.

## Appendix D

**Table A3:** Breakdown of the 402 articles that did not meet full-text screening criteria for articles that described integrated surveillance in Circumpolar Arctic and Subarctic regions, and associated reasons for exclusion.

Reason(s) for Exclusion	Number of Articles Excluded
Not an empirical research study published in English between 2005–2016	4
Main site or focus is not an Arctic or Subarctic region of the Circumpolar North	203
No considerations or implications for the natural environment	6
No considerations or implications for human health	15
No considerations or implications for surveillance	17
Not an empirical research study published in English between 2000–2016, *and* main site/focus is not an Arctic or Subarctic region	4
Not an empirical research study published in English between 2000–2016, *and* main site/focus is not an Arctic or Subarctic region, *and* no considerations or implications for surveillance	2
Not an empirical research study published in English between 2000–2016, *and* main site/focus is not an Arctic or Subarctic region, *and* no considerations or implications for human health	1
Main site or focus is not an Arctic or Subarctic region, *and* no considerations or implications for the natural environment	20
Main site or focus is not an Arctic or Subarctic region, *and* no considerations or implications for the natural environment *or* surveillance	14
Main site or focus is not an Arctic or Subarctic region, *and* no considerations or implications for the natural environment *or* human health	1
Main site or focus is not an Arctic or Subarctic region, *and* no considerations or implications for human health	20
Main site or focus is not an Arctic or Subarctic region, *and* no considerations or implications for surveillance	75
Main site or focus is not an Arctic or Subarctic region, *and* no considerations or implications for human health *or* surveillance	18
No considerations or implications for human health *or* surveillance	2
Total number of articles excluded	402

## Appendix E

**Table A4:** Citations and corresponding Arctic or Subarctic region(s) of focus for each of the 85 included articles, comprised of articles that informed, reviewed, and/or recommended integrated surveillance, and articles that focused on the development, implementation, and/or application of integrated surveillance systems.

Citation	Main Arctic or Subarctic Region of Focus
Articles Informing, Reviewing, and/or Evaluating Integrated Surveillance	
Abass et al. [94]	Circumpolar-wide
Amstislavski et al. [99]	Kanin Peninsula (Russia)
Andrachuk and Smit [130]	Tuktoyaktuk, NWT, Canada
Austin et al. [68]	Northern Canada
Banfield and Jardine [66]	Yellowknife, Northwest Territories (Canada)
Berner et al. [90]	Circumpolar-wide
Bhatia and Wernham [104]	North Slope, Alaska (USA)
Bond et al. [83]	Newfoundland and Labrador (Canada)
Bronson and Noble [122]	Northern Canada
Brubaker et al. [113]	Alaska (USA)
Brubaker et al. [121]	Northwest Alaska (USA)
Burger [58]	Amchitka Island, Aleutian Chain, Alaska (USA)
Byrne et al. [93]	St. Lawrence Island, Alaska (USA)
Ding et al. [131]	Finland, Iceland, Norway, and Sweden
Donaldson et al. [60]	Northern Canada
Donaldson et al. [95]	Circumpolar-wide
Dubé et al. [81]	Yukon River Basin (Canada and USA)
Ford et al. [123]	Northern Canada
Ford et al. [71]	Pan-Circumpolar
Ford et al. [79]	Nunavut, Nunavik, and Nunatsiavut (Canada)
Ford et al. [126]	Northern Canada
Furgal and Seguin [72]	Northern Canada
Gadamus [78]	Bering Strait Region, Alaska (USA)
Gibson et al. [102]	Pan-Arctic
Gunnarsdóttir et al. [116]	Iceland
Harley et al. [127]	Prudhoe Bay and Fairbanks, Alaska (USA)
Hori et al. [69]	James Bay Region, Northern Ontario (Canada)
Hueffer et al. [61]	Alaska (USA)
Kirk et al. [111]	Beaufort Sea, Alaska (USA)
Konkel [132]	Alaska (USA)
Kraemer et al. [59]	Circumpolar-wide
Krzyzanowski [74]	Northeastern British Columbia (Canada)
Kwiatkowski [114]	Northern Canada
Lepak et al. [98]	Northwestern British Columbia (Canada)
Lynn et al. [128]	Alaska (USA)
Martin et al. [115]	Nunavik, Northern Quebec (Canada)
McClymont Peace and Myers [105]	Northern Canada
Metcalf and Robards [117]	Bering and Chukchi seas, Alaska (USA)
Moiseenko et al. [124]	Lake Imandra watershed, Murmansk Oblast (Russia)
Natalia [118]	Circumpolar-wide
Noble and Bronson [129]	Northern Saskatchewan; Northwest Territories; and Voisey’s Bay, Northern Labrador (Canada)
Noble and Bronson [112]	Northern Canada
Parkinson [63]	Circumpolar-wide
Parkinson and Butler [82]	Circumpolar-wide
Pearce et al. [119]	Inuvialuit Settlement Region, Northwest Territories (Canada)
Pearce et al. [125]	Ulukhaktok, Northwest Territories (Canada)
Pennesi et al. [62]	Iqaluit, Nunavut (Canada)
Provencher et al. [97]	Northern Canada
Rosa et al. [133]	Barrow, Wainwright and Kaktovik, Alaska (USA)
Smith et al. [120]	Northern Canada
Tomaselli et al. [77]	Victoria Island, Nunavut (Canada)
Tsuji et al. [64]	James Bay Region, Northern Quebec (Canada)
Van Oostdam et al. [101]	Northern Canada
Wernham [65]	North Slope Borough, Alaska (USA)
Young et al. [91]	Circumpolar-wide
**Articles that Focused on Developing, Implementing, and/or Applying Integrated Surveillance**	
Berkes et al. [106]	Several regions across Northern Canada, including: the Beaufort Sea, Inuvialuit Settlement Region; Hudson Bay and James Bay regions of Northern Ontario and Quebec
Brook et al. [109]	Sahtu Settlement Area, Northwest Territories (Canada)
Bruden et al. [88]	Yukon-Kuskokwim Delta region, Alaska (USA)
Burger [58]	Amchitka Island, Aleutian Chain, Alaska (USA)
Burger and Gochfeld [107]	Adak Island, Aleutian Chain, Alaska (USA)
Burger et al. [141]	Aleutian Chain, Alaska (USA)
Dagsson-Waldhauserova et al. [137]	Iceland
Do et al. [67]	Northwest Territories and Nunavut (Canada)
Driscoll et al. [85]	Ketchikan, Angoon, Healy, Anderson, Cantwell, Point Hope, Kivalina, and Noatak, Nenana, Klawock, and Craig, Alaska (USA)
Driscoll et al. [84]	Ketchikan, Angoon, Healy, Anderson, Cantwell, Point Hope, Kivalina, and Noatak, Alaska (USA)
Dudarev et al. [136]	Select regions of the Russian Arctic, Siberia, and Far East
Dunlap et al. [110]	Yukon River, Alaska (USA)
Fall [135]	Alaska (USA)
Ford et al. [108]	Iqaluit, Nunavut (Canada)
Germain [87]	Blanc-Sablon, Lower North Shore of the St. Lawrence River and Kangiqsualujjuaq, Nunavik, Northern Quebec (Canada)
Gunnarsdóttir et al. [75]	Iceland
Huntington et al. [139]	Bering Strait Region (Russia and USA)
Khalil et al. [80]	Northern Sweden
Laidler et al. [70]	Cape Dorset, Igloolik, and Pangnirtung, Nunavut (Canada)
Larrat et al. [92]	Nunavik, Northern Quebec (Canada)
Ludwicki et al. [142]	Greenland
Miller et al. [86]	St. Lawrence Island, Alaska (USA)
Montrose et al. [73]	Fairbanks, Alaska (USA)
Nilsson et al. [76]	Circumpolar-wide
Pacyna et al. [103]	Circumpolar-wide
Pardhan-Ali et al. [138]	Northwest Territories (Canada)
Skandfer et al. [140]	Murmansk Oblast (Russia)
Tremblay et al. [89]	Nunavik, Northern Quebec (Canada)
Vlasova and Volkov [100]	Murmansk, Arkhagelsk Oblast, and Republic of Komi (Russia)
Wesche et al. [134]	Old Crow, Yukon (Canada)

## Appendix F

**Table A5:** Enabling components for integrated surveillance systems in Circumpolar Arctic and Subarctic regions as described or recommended by the literature.

	Component	Definition	Example of Linkages to Surveillance Attributes and/or Activities
**Structural**	Decision-making and accountability structures	Procedures for guiding decision-making related to surveillance information, and structures for holding decision-makers accountable for their actions	In the case of surveillance information that Germain [87] used to inform early-warnings of avalanche risks in Quebec, bottom-up approaches, in this context, were largely ineffective. Instead, top-down approaches from the municipal and provincial government were recommended.
	Enabling regulatory, policy, and funding environments	Resources, personnel, and other forms of support that provide or enhance enabling environments for surveillance activities	The International Circumpolar Surveillance system, as described by Parkinson [63] allowed for the collection and sharing of uniform laboratory and epidemiologic data between Arctic countries, and assisted in the development of coordinated prevention and control strategies.
	Existing surveillance systems and/or data	Use of existing surveillance systems and data that focus on environment and/or health issues of interest	Pacyna et al. [103] described how assessment tools developed for the EU ArcRisk project were enhanced through use of existing databases, models, and monitoring systems from the Arctic Monitoring and Assessment Program.
	Technologies and tools	Technological tools for capturing surveillance data, such as remote-sensing, Geographic Information Systems, and satellite imagery	Satellite imagery was one of many useful tools described by Laidler et al. [70] that Inuit in Iqaluit, Nunavut draw upon and used alongside experiential knowledge in monitoring sea ice dynamics.
**Processual**	Capacity to adapt or adjust surveillance activities	Capacity to adapt the processes and approaches to certain surveillance activities to allow for a system to be more responsive to changing environmental conditions as well as the needs of stakeholders and/or end-users	The iterative development of metrics, a survey instrument, and a protocol for collecting sentinel surveillance data on the health effects of climate change in Alaska by Driscoll et al. [84,85] led to targeted climate change adaptation strategies that were both locally-determined and data-driven.
	Involving distinct knowledge systems and disciplines	Capacity to involve multiple distinct knowledge systems and sources—such as local and Indigenous knowledge, and/or multiple disciplinary perspectives—in various stages of developing, implementing, and/or using surveillance systems	Tremblay et al. [89] explained how bringing together Indigenous and Western knowledge systems surrounding sea ice dynamics and climate change in Nunavik informed adaptation strategies for accessing land and resources that were grounded in more holistic understandings of environmental changes that respected Indigenous perspectives and worldviews.
	Involving multiple sources and types of surveillance data	Use of multiple approaches to surveillance data collection and analysis (e.g., qualitative and quantitative) appropriate for the specific purpose(s) and use(s) of a surveillance system	In a Northern environmental impact assessment described by Bronson and Noble [122], quantitative data from mail-out surveys were supplemented with qualitative data from semi-structured interviews with stakeholders to provide additional data and understandings for identifying and monitoring human health determinants.
**Relational**	Capacity to recognize and meet stakeholder needs	Ability to involve stakeholders in decision-making processes related to the development and implementation of integrated surveillance systems	The main goals of the Nunavik Trichinellosis Prevention Program described by Larrat et al. [92] were to prevent human trichinellosis and indirectly participate in the public health mission of ensuring health by increasing food diversity and positively impacting economic, social, and cultural role of the walrus hunt.
	Communication strategies	Clear, consistent communication procedures and channels that allow for stakeholders to voice potential ideas or concerns in a timely manner	Communication about resource development projects was recommended by Banfield and Jardine [66] to be kept at the forefront in all consultations, decision-making processes, and long-term monitoring efforts to facilitate relevant public health promotion through ongoing information-sharing, reporting of on-site monitoring activities, and educational initiatives.
	Strategies for managing collaborations involving multiple stakeholders	Efforts to encourage and sustain respectful and mutually-supportive collaboration among stakeholders involved in various stages of surveillance system development and implementation.	Collaboration among stakeholders helped to develop the community-based monitoring program described by Brook et al. [109] that provided more locally-relevant and useful information, ensured higher levels of support from all those involved, as well as functioned as a form of peer-review throughout the development process.
	Strategies for engaging specific, key stakeholders	Efforts to identify and reach out to key individuals and groups to identify and address common goals as well as potential challenges related to integrated surveillance	Driscoll et al. [84] discussed how consultations with village and tribal administrators, community members, as well as with an international team of climate change researchers helped the research team identify categories of exposure to the environmental effects of climate change, measurable health outcomes, as well terminology to use in a survey tool as part of a community-based sentinel surveillance system in Alaska.
	Training and educational opportunities	Provision of training or educational opportunities to key individuals and groups that strengthen or enhance approaches for collection, analyses, and interpretation of environment and health data	Hueffer et al. [61] recommended that additional resources and training were needed to ensure adequate numbers of trained staff were available to address the emerging public and wildlife health impacts posed by climate change in Alaska, and enhance the capacity to monitor those potentially climate-sensitive infections that are most likely to have a large public health impact.

Note: Corresponding examples of each component with linkages to surveillance attributes and activities are provided, as identified in the 85 articles included for review.
